# In Silico Study of 17‐DMAG Derivatives: Integrating QSAR, Molecular Docking, Molecular Dynamics, and ADME Analysis

**DOI:** 10.1155/tswj/1136652

**Published:** 2026-06-17

**Authors:** Víctor Eljach-Cordoba, Maicol Ahumedo-Monterrosa, Jhoan Piermattey-Ditta, Jairo Mercado-Camargo, Jorge Anaya-Gil

**Affiliations:** ^1^ Natural Products Group, School of Pharmaceutical Sciences, Zaragocilla Campus, University of Cartagena, Cartagena, 130015, Colombia, unicartagena.edu.co

**Keywords:** ADME, geldanamycin, Hsp90, molecular dynamics, QSAR

## Abstract

The Hsp90 protein is crucial for cancer cell survival by regulating essential processes such as cell division, proteostasis, and the correct folding of proteins, including transcriptional factors, growth factors, and kinases. Due to its therapeutic relevance, Hsp90 has become a target for developing anticancer drugs. In this in silico study, a 2D‐QSAR analysis was performed with geldanamycin derivatives, 33 of these selected for the training set, obtaining a model with predictive parameters (*R* ^2^ = 0.710, *Q* ^2^ = 0.626, *F* = 17.060). The test set of 10 compounds reached an external *R* ^2^ of 0.610. Additionally, a molecular docking between geldanamycin derivatives and Hsp90 was carried out using the AutoDock4.2 program; the best candidates were selected according to their binding energy and ligand efficacy to form complexes, and the interactions were analyzed.

The protein–ligand complexes with the best binding energy were subjected to 100‐ns molecular dynamics simulations to evaluate the stability of the complexes as a function of time, and the binding free energies were calculated for each complex. Finally, an ADME analysis was performed using SwissADME, allowing the design of new ligands with improved binding affinity and optimized pharmacokinetic properties compared to their predecessors for developing Hsp90 inhibitors.

## 1. Introduction

Heat shock protein 90 (Hsp90) is a chaperone protein abundant in prokaryotes and eukaryotes and is believed to influence significantly different types of cancer, such as breast, lung, and prostate cancer [1, 2]. Its main function is maintaining proteostasis, apoptosis, cell signaling, and protein folding [3]. Its inhibition prevents its association with these proteins, which causes them not to fold correctly and is degraded by mechanisms mediated by ubiquitin [4]. Therefore, Hsp90 plays a fundamental role in the survival and proliferation of healthy and tumor cells. For this reason, it has become a significant biological target for antagonists such as geldanamycin (GDM) and its derivatives [5]. However, this has been overlooked in some fields of medicine with respect to the development of new analogs since Hsp90 is also found at considerable levels in healthy cells [6, 7].

Several studies have determined the differences between the characteristics of Hsp90 in cancerous and healthy cells, which makes it a viable biological target [8]. For example, in different types of cancer, this protein has been highly expressed, up to 2 to 10 times higher than the levels found in healthy cells under conditions of high temperature, abnormal pH, and lack of nutrients [9]. In addition, the function of Hsp90 is regulated by co‐chaperone proteins and post‐translational modifications. Hsp90 contains many post‐translational sites where modifications can occur, allowing a notable differentiation between the proteins of both types of cells [5]. Another essential aspect implies that the Hsp90 proteins of cancer cells are more susceptible to inhibitors because the complexes enhance their activity as an ATPase, they form with co‐chaperones, which favors the binding of antagonist molecules [1, 6]. Structurally, Hsp90 has 3 known domains: the N‐terminal domain that serves as the active site for ATP and protein antagonists, the intermediate domain that is responsible for binding to client proteins, and the C‐terminal domain that is responsible for protein dimerization [10].

Different compounds have been evaluated and proposed as Hsp90 antagonists, including GDM, 17‐AAG, and 17‐DMAG. Clinical studies have shown little effective inhibitory concentrations and considerable toxicity [11]. Therefore, it is essential to search for and design drugs that contribute to specialized therapies that show low toxicity and excellent solubility in clinical trials [12]. Due to the high cost of experimental studies of derivative synthesis and *in vitro* tests with cell lines, it is necessary to apply other alternatives for the optimization and search for new antagonists. Therefore, in this work, different methodologies of computational chemistry are sequentially applied, such as 2D‐QSAR, molecular docking, molecular dynamics, and analysis of absorption, distribution, metabolism (biotransformation), and excretion (ADME) properties to propose 17‐DMAG analogs with better pharmacokinetic characteristics, expand the amount of computational data, and complement them with experimental data for the proposal of new analogs [13, 14].

## 2. Methodology

### 2.1. 2D‐QSAR

#### 2.1.1. Data Set

The chemical structures of the 17‐DMAG analogs and their *in vitro* biological activity (IC_50_ values) were taken from experimental works [14, 15]. They were built using the GaussView 6.0 program [16]. These molecules were optimized in their geometry using the PM6 semiempirical method [17]. Geometry optimizations were carried out with the Gaussian16 suite of programs [18], and descriptors such as energy, HOMO, LUMO, and dipole moment were calculated.

#### 2.1.2. Compound Classification and Descriptor Calculation

For constructing the 2D‐QSAR model, 42 compounds were taken from 17‐DMAG (17‐dimethylaminoethylamino) with their biological activity [14] and 17‐DMAG. These 43 compounds were divided into two sets by randomization methods. A training set included 33 compounds, as shown in Table 1, while the test set included 10. Of the latter, 6 were taken from the base article and four were taken from an analogous study of GDM derivatives applied to the same cell line [15]. Topological, structural, and physicochemical molecular descriptors were calculated using the MOE program library, obtaining 209 descriptors [19, 20]. In addition, descriptors generated in the Gaussian output during the optimization process were calculated. For constructing the models using combinatorial methods, 47 filtered descriptors were used from 213 descriptors calculated in the MOE and Gaussian programs.

**TABLE 1:** Analogs of 17‐(dimethylaminoethylamino) were used in the training set with modifications at carbon 11 (R_11_) and carbon 17 (R_17_).


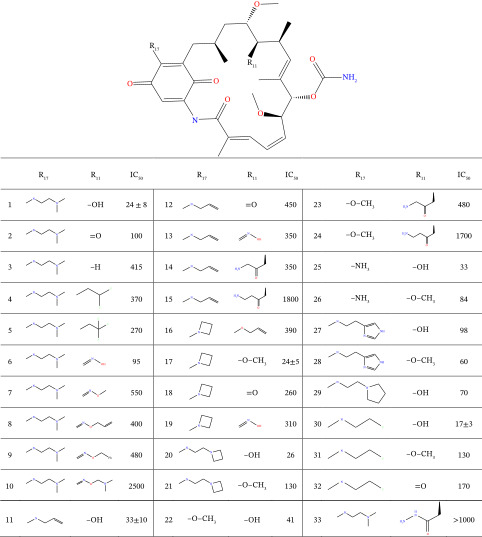

#### 2.1.3. Descriptor Selection and Model Development

The calculated descriptors were filtered using correlation matrices, and those descriptors that showed a high correlation with each other, with a Pearson correlation coefficient greater than 0.6, were eliminated. After the filter, the most relevant descriptors were subjected to multivariate regression methods (MLR) to obtain QSAR models that explain and predict the activity of the proposed compounds. For this purpose, combinatorial methods were used with MLR to generate all possible models with the filtered descriptors [21].

#### 2.1.4. Internal and External Validation of the Model

Various model validation tests were performed to confirm the robustness and predictive capacity of the obtained models, where several key statistical parameters were calculated [20, 22]. These parameters include the internal *R* ^2^, which measures the degree of fit of the model with the data of the training set (Equation (1)); the adjusted internal correlation coefficient (R^2^_adj_), which adjusts the *R* ^2^ considering the number of predictor variables; and the sample size for a more precise assessment of the model fit (Equation (2)). The cross‐validation coefficient (*Q* ^2^) was also calculated, which evaluates the capacity of the model to predict new data through cross‐validation (Equation (3)).

(1) R2=1−∑i=1nYobs−Ycalc2∑i=1nYobs−Y¯calc2.

(2) Radj2=n−1×R2−pn−1−p.

(3) Q2=1−∑Yobs/train−Ycalc/train2∑Yobs/train−Y¯calc/train2.

The Fisher test (F) was used to analyze the significance of the regression coefficients; the predictor variables have a significant impact if the F value is high for a *p*‐value < 0.05 (Equation (4)). In addition, the standard error (*s*) was calculated, which should be low for a good model (Equation (5)), the sum of the squares of the prediction error (PRESS), which should have a low value concerning the sum of the squares (SSY) corresponding to the dependent variable, and the PRESS/SSY ratio, which should be less than 0.4. On the other hand, the root mean square error (Equation (6)) and the root mean square error calibration (RMSEC) (Equation (7)) were calculated, and these last two should be low for a good model. All parameters contribute to a comprehensive understanding of the accuracy and reliability of the predictive model:

(4) F=N−p−1×R2p×1−R2.

(5) s=∑Yobs−Ycal2N−p−1 .

(6) MSE=1n∑i=1nYobs−Ycalc2.

(7) RMSEC=∑Yobs−Ycal2n .

An external validation is performed to evaluate the predictive ability of the model on compounds that are not part of the training set, in order to avoid false positives or overfitting. In this study, several external validation metrics were taken into account [21], such as the external validation coefficient (external *R* ^2^), which measures the accuracy of the model when predicting external data, and the external validation coefficient for the equation with the intercept equal to 0 ($R_{0}^{2}$).

Additionally, the indicator (*R* ^2^‐$R_{0}^{2}$)/*R* ^2^, which evaluates the robustness of the model, and the coefficient *k* for the equation with the intercept equal to 0, which verifies the consistency of the predictions, were considered.

Other validation parameters are based on the criteria of Golbraikh and Tropsha [23], which are used to measure the predictability of a model by external testing. According to these criteria, the square of the lines drawn with zero intercept ($r_{0}^{2}$) correlation coefficient should be close to the *R* ^2^ value for the developed model (Equation (8)). On the other hand, both *Q* ^2^ and *R* ^2^ rely on the average of the activities of the compounds used for the training set. Therefore, high *R* ^2^ values can be obtained when the test set has a wide range of information regarding the activity. However, this does not indicate that the predicted activity is close to the corresponding observed values. To verify the closeness between the observed and predicted values, $r_{m}^{2}$ is used (Equation (9)):

(8) r02=1−∑i=1nYobs−k×Ycalc2∑i=1nYobs−Y¯obs2,

(9) rm2=r2×1−r2−r0′2.

#### 2.1.5. Domain of Applicability

Principal component analysis (PCA) was used to delimit the chemical space. The method converts the original data into an orthogonal coordinate system (PC) that corrects the correlations between descriptors and maximizes the variation of the compounds [24]. The applicability domain (AD) is defined by the PCA score plot, where the boundary formed by the training set compounds establishes the AD.

### 2.2. Molecular Docking

#### 2.2.1. Molecular Docking Validation

The 3D structure of the protein released from the Protein Data Bank (PDB) with PDB code 1OSF in complex with the co‐crystallized ligand 17‐DMAG. The complex and the ligand were separated using the Chimera software [25] for their subsequent preparation, separately for docking with the AutoDockTools program [26]. The co‐crystallized ligand and the derived compounds were prepared by adding hydrogens to the structure, and Gasteiger charges and nonpolar hydrogens were removed. For protein preparation, polar hydrogens were added, residues were repaired, and Kollmann charges were added.

The validation was performed in triplicate in the AutoDock 4.2 program, where the grid box was adjusted with dimensions (40 × 40 × 40 Å) with a distance of 0.375 Å based on the binding site of the co‐crystallized ligand reported in the literature (X, Y, and Z coordinates) [27]. For the docking, the genetic algorithm with 200 poses was used; the other parameters were taken from [28]. Once the docking was done, the best poses were chosen to calculate the root mean square deviation (RMSD) concerning the coordinates of the atoms of the ligand co‐crystallized with the protein; for this, the Chimera software was used.

### 2.3. Molecular Dynamics Simulations

The protein–ligand complexes that showed the best affinity energy in molecular docking were subjected to 100‐ns molecular dynamics simulations using GROMACS software Version 2020.2 [29] to evaluate their stability throughout the simulation. The force field used for the protein and ligand was the CHARMM force field [30] and the CHARMM general force field (CGenFF) [31], respectively. The complexes were placed in a cubic periodic box, and each complex was solvated with TIP3P‐type water molecules under periodic boundary conditions [32]. The system was neutralized, and the medium’s ionic strength (0.1 mol/L) was adjusted by adding Na^+^ and Cl^-^ ions while keeping the number of particles constant. Minimization of the systems was performed until convergence was reached. Phase equilibration was then performed, keeping constant pressure and temperature (NVT and NPT) at 300 K and 1.0 bar, respectively. The equilibration periods lasted 1.0 ns. The production duration was 100 ns, and trajectories were saved every 0.01 ns. The molecular dynamics results were used to calculate the RMSD, root mean square fluctuations (RMSF), hydrogen bonds (H‐bonds), and radius of gyration, which were determined to analyze the flexibility and stability of the complex over time. The MMPBSA.py script [33] in the AMBER 21 suite predicted the binding free energies between the protein complexes. To apply the MMPBSA.py script, the topology, coordinates, and production files generated by GROMACS were converted to their AMBER counterpart. The interaction energy and solvation‐free energy for the complex, receptor, and ligand were used to estimate the free energy according to the methods used in MM/GBSA [34].

### 2.4. ADME Properties Analysis

GDM and its derivative 17‐DMAG were taken as a reference point for analyzing ADME pharmacokinetic properties. In addition, the 3 proposed molecules were taken from the QSAR model to estimate their ADME properties on the SwissADME server [35].

## 3. Results

### 3.1. 2D‐QSAR

#### 3.1.1. Model Generation and Validation

Two models were chosen to carry out the internal validation tests, represented by Equations (10) and (11). The parameters calculated for each model are presented in Table 2. To determine the fit capacity of the models, the squared regression coefficient (*R* ^2^) is considered first, which, according to the literature, should be greater than 0.6 [22]. In this case, Model 1 presented an *R* ^2^ of 0.710, indicating a good fit, while Model 2 reached an *R* ^2^ of 0.631, falling within the acceptable parameters for a predictive model. On the other hand, low values were obtained for the PRESS and high values for the SSY of the observed values with respect to the mean. Furthermore, the relationship between the two (PRESS/SSY) was less than 0.4, evidencing good predictive quality. The adjusted coefficient of determination (R^2^_a_) was also calculated; the R^2^_a_ was 0.667 for Model 1, and 0.578 for Model 2. This suggests that the first model does not experience a significant decrease in its explanatory capacity when incorporating a new descriptor. In contrast, Model 2 could suffer a more significant decrease:

(10) pIC50=58.661112.489.43040.90640.09653−BCUTSMR3−QVSAFHYD−bdouble+HOMO,

(11) pIC50=5.595410.844.3981.5922.952−b1rotR+PEOEVSAFPOS+SlogPVSA0−SMRVSA4.

**TABLE 2:** Training set statistical parameters.

Statistical parameters	M_1_	M_2_
n	33	33
*R* ^2^	0.710	0.631
*R* ^2^_adj_	0.667	0.578
s	0.329	0.464
F	17.060	11.951
*p*	< 0.0001	< 0.0001
*Q* ^2^	0.626	0.422
SSY	10.439	16.341
PRESS	3.027	6.030
PRESS/SSY	0.290	0.369
MSE	0.0917	0.1827
RMSEC	0.3029	0.4275

For overall significance to exist, the F value of the model must have a *p*‐value < 0.05, which rejects the null hypothesis that there is no significance in the regression model. A higher F indicates greater importance for the multiple regression coefficient. In this case, the F value was 17.060 for Model 1 and 11.951 for Model 2, confirming that both models are significant regarding their regression coefficients. In addition, the standard error was evaluated, obtaining a value of 0.329 for the first model and 0.464 for the second model. Another critical parameter is the cross‐validation coefficient (Q^2^), which must be greater than 0.5 to ensure good predictive capacity [22]. Model 1 presented a Q^2^ of 0.626 in this analysis, indicating the model’s good predictive capacity and reliability. In contrast, Model 2 obtained a Q^2^ of 0.464.

Given the results of both models, Model 1 was chosen because it presented better parameters than Model 2. In addition, the cross‐validation test of Model 2 did not reach the minimum threshold of 0.5, as reported in the literature.

**FIGURE 1:**
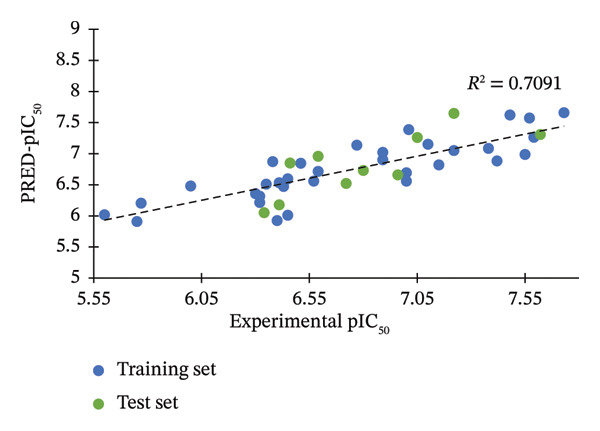
Experimental biological activity (pIC_50_) and predicted activity (PRED‐pIC_50_) for the training and test set.

Model 1 (Figure 1) was built from the descriptors, as shown in Tables 3 and 4, the descriptor with the largest contribution in the equation being BCUT_SMR_3, which is related to molar refractivity, a measure of the polarizability and volume of a molecule. Second place is Q_VSA_FHYD, which refers to the van der Waals fractional hydrophobic surface area, indicating the proportion of the hydrophobic molecular surface. The third important descriptor is b_double, quantifying the number of carbon atoms with double bonds or in the sp^2^ hybridization state. Finally, the HOMO, the highest energy‐occupied orbital, is crucial in determining chemical reactivity, as it represents the highest electron‐containing energy level in a molecule.

**TABLE 3:** Calculated parameters for the training set.

Compounds	pIC_50exp_	pIC_50pred_	BCUT_SMR_3	Q_VSA_FHYD	b_double	HOMO (ev)
1	7.57	7.57	2.969	0.608	9	−1.480
2	7.00	6.56	2.925	0.599	10	−9.256
3	6.38	6.87	2.947	0.637	9	−8.826
4	6.43	6.48	2.999	0.609	9	−8.951
5	6.57	6.56	2.999	0.599	9	−9.034
6	7.00	6.69	2.928	0.584	10	−8.919
7	6.30	6.36	2.928	0.619	10	−8.912
8	6.40	5.92	2.928	0.569	11	−8.915
9	6.32	6.22	2.928	0.635	10	−8.872
10	5.60	6.02	2.928	0.658	10	−8.627
11	7.42	6.88	2.954	0.531	10	−8.762
12	6.35	6.51	2.924	0.513	11	−8.867
13	6.45	6.60	2.927	0.499	11	−8.911
14	6.45	6.01	2.960	0.518	11	−8.974
15	5.75	5.91	2.960	0.530	11	−8.738
16	6.41	6.53	3.042	0.550	9	−8.542
17	7.55	6.99	2.954	0.616	9	−8.814
18	6.59	6.72	2.954	0.549	10	−8.674
19	6.51	6.85	2.955	0.534	10	−8.708
20	7.59	7.27	2.967	0.569	9	−8.754
21	6.89	7.02	2.960	0.600	9	−9.270
22	7.38	7.09	2.973	0.570	9	−9.720
23	6.32	6.32	2.959	0.575	10	−9.703
24	5.77	6.21	2.960	0.586	10	−9.700
25	7.48	7.62	2.953	0.544	9	−9.296
26	7.10	7.15	2.959	0.588	9	−9.161
27	7.01	7.39	2.954	0.569	9	−9.250
28	7.22	7.05	2.960	0.600	9	−8.901
29	7.15	6.82	2.965	0.619	9	−8.750
30	7.73	7.66	2.998	0.578	8	−9.135
31	6.89	6.90	2.968	0.606	9	−8.770
32	6.77	7.14	2.924	0.541	10	−8.976
33	6.00	6.48	2.959	0.567	10	−8.799

*Note:* Experimental pIC_50_ and the pIC_50_ predicted from the calculated descriptors are shown for each training set compound.

**TABLE 4:** Calculated parameters for the test set.

Compounds	pIC_50exp_	pIC_50pred_	BCUT_SMR_3	Q_VSA_FHYD	b_double	HOMO
1	7.62	7.31	2.97	0.57	9	−8.559
2	7.22	7.65	2.95	0.54	9	−8.863
3	7.05	7.26	2.95	0.59	9	−8.913
4	6.96	6.66	2.96	0.54	10	−9.111
5	6.80	6.73	3.01	0.67	8	−8.724
6	6.72	6.52	2.98	0.54	10	−9.138
7	6.59	6.96	2.95	0.53	10	−8.906
8	6.46	6.85	2.95	0.53	10	−8.708
9	6.41	6.18	3.00	0.64	9	−8.902
10	6.34	6.05	2.98	0.68	9	−8.550

*Note:* The experimental and predicted pIC_50_ values are shown. In addition, the values of the molecular descriptors used for the test set are presented.

For external validation, the first parameter considered is the external *R* ^2^. In this case, the model presented an external *R* ^2^ of 0.610, as shown in Table 5, which shows a good correlation and confirms the model’s validity in predicting data not included in the training.

**TABLE 5:** Statistical parameters calculated for the test set.

Statistical parameters	M_1_
*R* ^2^_external_	0.610
*R* ^2^_0_	0.609
(*R* ^2^‐*R* ^2^_0_)/*R* ^2^	0.002
k	0.998
K’	1.0003
(*R* ^2^‐R^’ 2^_0_)/*R* ^2^	−0.390
*R* ^2^_m_	0.592

For the model, *k* and k’ values were obtained within the allowed range (0.85 ≤ *k* ≤ 1.15). Using the values of *k* and k’, the square of the correlation coefficient of the lines drawn with zero intercept (R^2^_0_ and R′^2^_0_) was calculated, which was quite close to the value of the external *R* ^2^. Finally, the value of r^2^_m_ was calculated, obtaining a value of 0.592.

#### 3.1.2. Domain of Applicability

**FIGURE 2:**
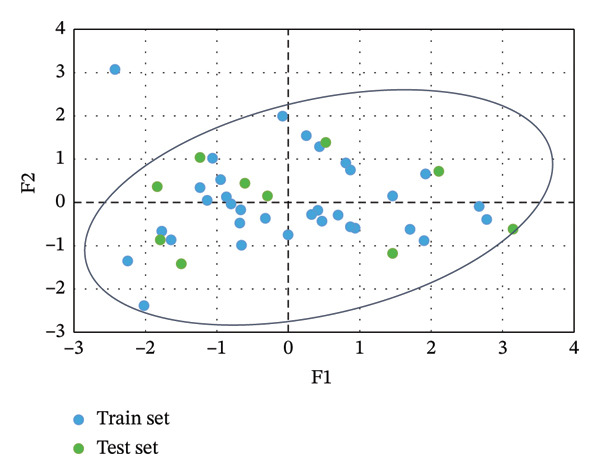
The applicability domain was analyzed using the PCA bounding box approach.

The training and test sets were subjected to a PCA to assess their distribution in chemical space. Figure 2 shows that the compounds in the test set are located within the edges of the training set. This proximity suggests that the QSAR model has a clearly defined domain of applicability since the new compounds are close to the regions where the model was previously validated.

#### 3.1.3. Calculation of Activities of Proposed Compounds

Three ligands were designed based on the QSAR model to optimize activity while preserving hydrogen bond donor groups at the C11 carbon substituents, as detailed in Table 6. Only ligand 1MP had an IC_50_ lower than the native ligand (24 nM). In contrast, for ligand 2MP, the predicted mean inhibitory concentration was 4 units higher than that of the native ligand. Meanwhile, ligand 3MP showed the highest inhibitory concentration, 14 units higher than the native ligand.

**TABLE 6:** Predicted activities for the proposed ligands.

Compounds	2D structure	IC_50_ (nM)
1MP	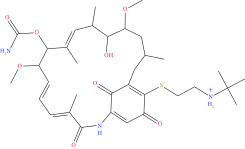	23.50
2MP	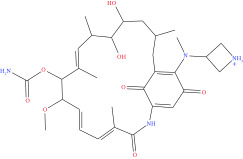	38.06
3MP	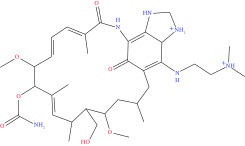	28.70

### 3.2. Molecular Docking

#### 3.2.1. Docking Validation

The re‐docking of the co‐crystallized ligand into the Hsp90 binding site was performed in triplicate. In each run, the most populated cluster was selected, choosing the lowest energy conformation, and the average energy value was ‐9.46 ± 0.7 kcal/mol with a ligand efficiency of ‐0.217 ± 0.02 and an RMSD of 1.095 ± 0.07 Å (Figure 3(a)), as detailed in Table S1.

FIGURE 3The co‐crystallized ligand (magenta) together with the re‐docked ligand for validation (cyan) in complex with the Hsp90 protein. (a). Interactions between the binding site and the co‐crystallized ligand (b). Interactions between the binding site and the re‐docked ligand (c).

**Figure:**
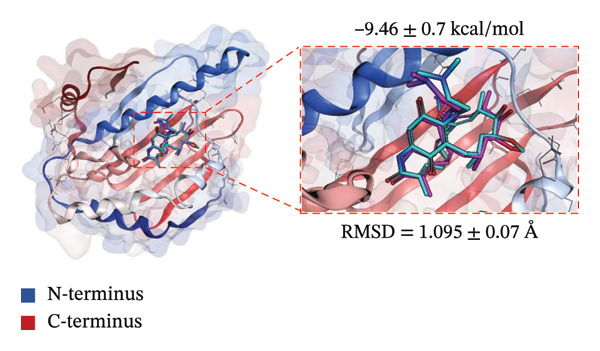
(a)

**Figure:**
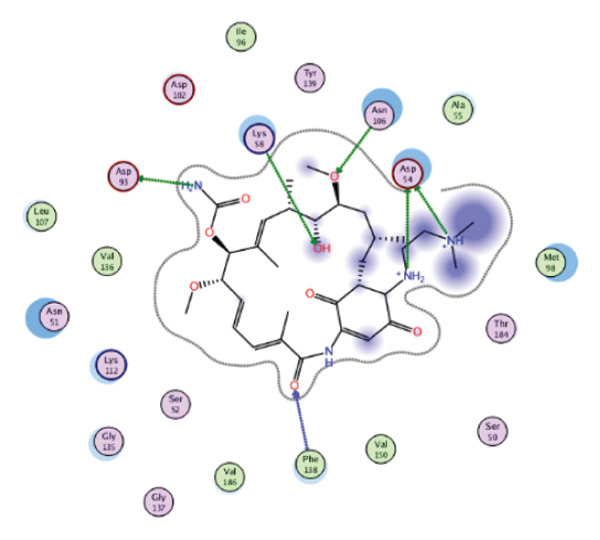
(b)

**Figure:**
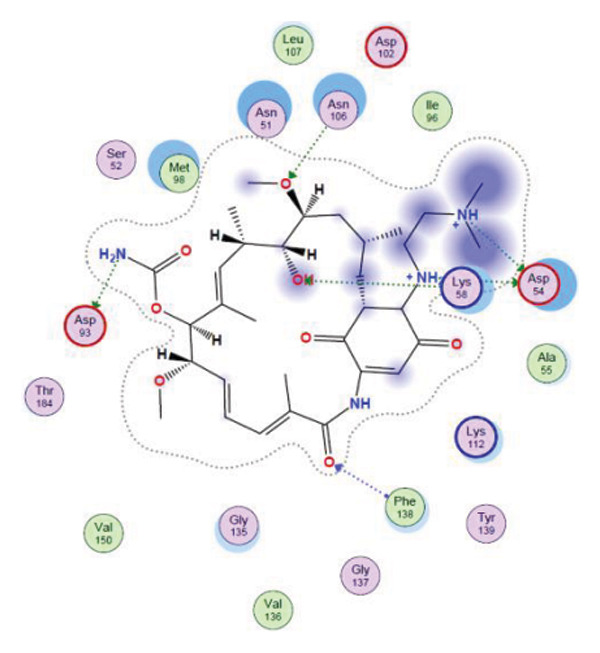
(c)

The hydrogen bonds of the co‐crystallized ligand, with the residues Lys58, Asp54, Asp93, Asn‐106, and Phe138 interact with the hydroxyl group at the C11 position, the side chain, and with the nitrogen atoms of the dimethylaminoethylamino group at the C17 position, the amino group of the carbamate at the C7 position, the oxygen of the methoxide group at the C12 position, and the amide group adjacent to the benzoquinone, respectively. In both cases, most of the interactions are reproduced in the crystal structure and in the re‐docked ligand (Figures 3(b), 3(c)).

#### 3.2.2. Docking of 17‐DMAG Derivatives

The results for the five ligands with the best binding energies are presented in Table 7 and Figure S1. Ligand 1g, with a binding energy of −7.81 kcal/mol, formed two hydrogen bonds. The first was established between the hydroxyl group at position C11 and the Met98 residue; the second was between the methoxide group at position C6 and the Lys112 residue (Figure 4(a)). Ligand 3g had a binding energy of −7.5 kcal/mol and formed four hydrogen bonds. One interaction between the tertiary amino group of the pyrrolidine ring at position C11 with the Asp93 residue, and another between the oxygen of the methoxide group at position C12 with the Met98 residue. The last two hydrogen bonds were formed between the oxygen of the methoxide group at the C6 position and residues Lys112 and Val136, respectively (Figure 4(b)).

**TABLE 7:** Molecular docking results for the top 5 ligands, showing mean inhibitory activity (IC_50_), binding energy, and ligand efficiency (EL).

Compounds	2D structure	IC_50_	Binding energy (kcal/mol)	EL
1g	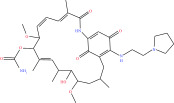	70	−7.81	−0.17
3g	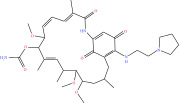	160	−7.5	−0.16
7f	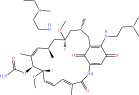	460	−7.32	−0.15
7a	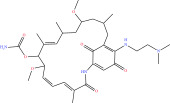	415	−7.28	−0.17
4d	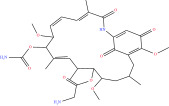	480	−7.25	−0.13

FIGURE 4Hydrogen bond interactions between the receptor and the top 5 ligands 1g (a), 3g (b), 7f (c), 7a (d), 4d (e).

**Figure:**
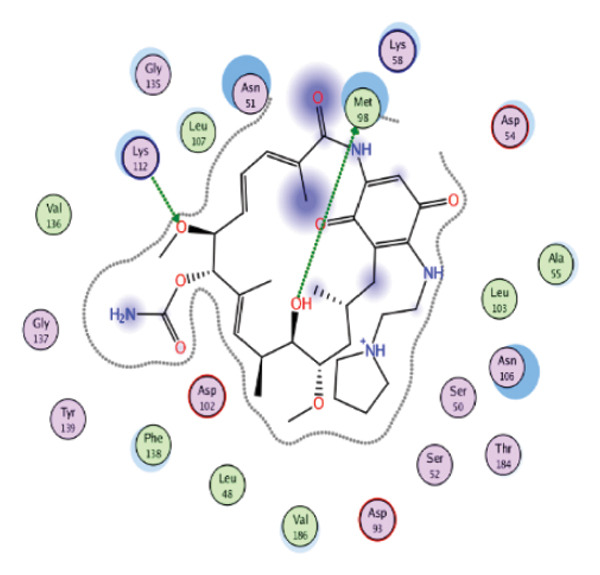
(a)

**Figure:**
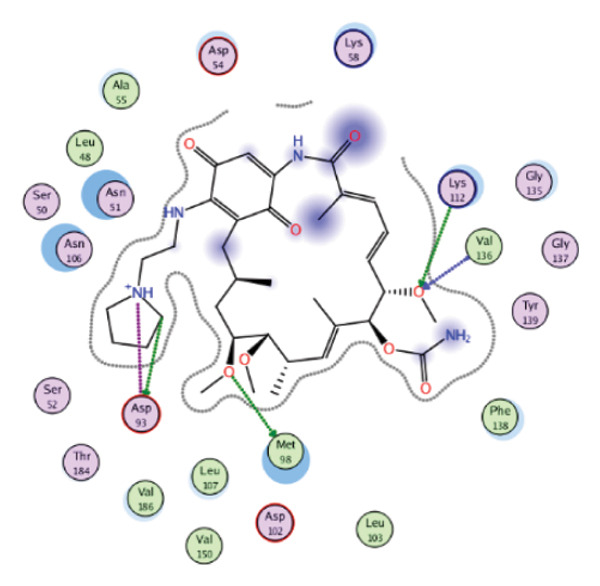
(b)

**Figure:**
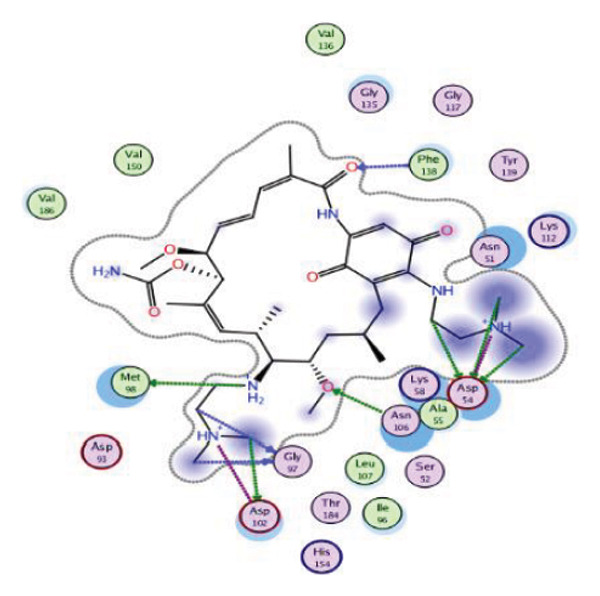
(c)

**Figure:**
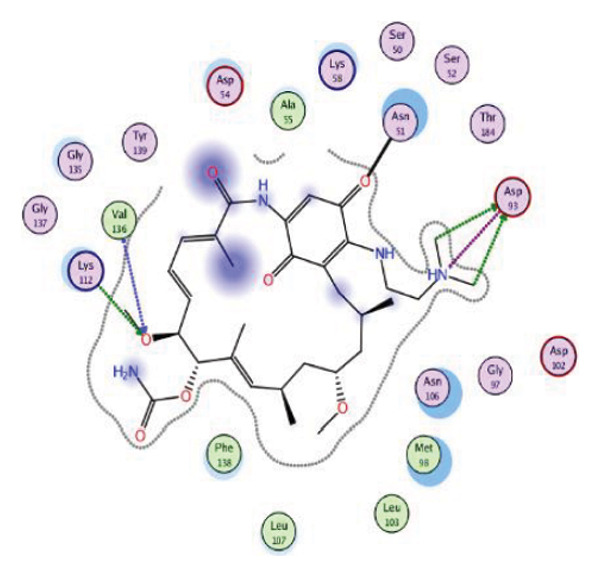
(d)

**Figure:**
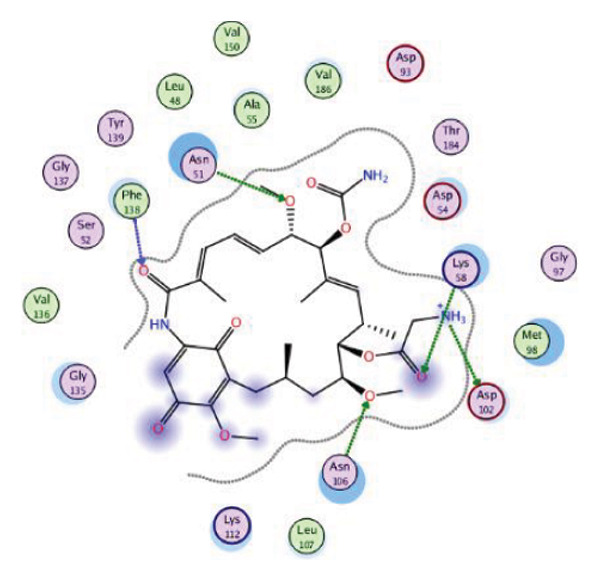
(e)

Ligands 1g and 3g have the same substituent (2‐(pyrrolidine‐1‐yl)‐ethan‐1‐amine) at position C17. On the other hand, ligand 7f, with a binding energy of −7.32 kcal/mol, forms four hydrogen bonds. Between the tertiary amine of the substituent (dimethylethane‐1,2‐diamine) at position C17 and the Asp54 residue, the secondary amine of the substituent at position C11 and the Met98 residue; and the third, in the same way, the tertiary amine of the substituent at position C11 and the Asp102 residue. On the other hand, the oxygen of the methoxide group at position C12 interacted with the Asn106 residue. Finally, the hydrogen bond between the oxygen of the carbonyl group at position C1 and Phe138 (Figure 4(c)).

Ligand 7a, whose binding energy was −7.28 kcal/mol, forms 4 hydrogen bonds. The protonated tertiary amine of the substituent at position C17 and the Asp93 residue, between the carbonyl group of the benzoquinone at position C18 and the Lys112 residue, between the oxygen methoxide group at position C6 and the Val136 and Lys112 residues (Figure 4(d)). Ligands 7a and 7f also had the same substituents at position C17; the only difference was the substituent at position C11. On the other hand, ligand 4d had a binding energy of −7.25 kcal/mol and formed 5 hydrogen bonds. The first one occurred between the oxygen of the methoxide group at position C6 and the Asp51 residue; the second one occurred between the carbonyl group of the substituent at position C11 and the Lys58 residue; the third occurred between the primary amine of the substituent in position C11 and the Asp102 residue; the fourth occurred between the oxygen of the methoxide group in position C12 and the Asn106 residue; the fifth occurred between the oxygen of the carbonyl group in position C1 and the Phe138 residue (Figure 4(e)), and this did not present an analog with a common substituent like the previous ones.

#### 3.2.3. Docking of the Proposed Ligands

The proposed ligands were docked from the QSAR model, obtaining their binding energies and efficiencies, as detailed in Table S2. Ligand 1MP presented a binding energy of −6.35 kcal/mol, forming four hydrogen bonds. The first was established between the carbonyl group of the benzoquinone at position C18 and the Lys58 residue; the second, with the carbonyl group of the benzoquinone at position C20; the third, between the protonated secondary amine at position C17 and the Asp102 residue; and the fourth, between the carbonyl of the carbamate group at position C7 and the Lys112 residue (Figure 5(a)). Ligand 2MP showed a binding energy of −7.15 kcal/mol, forming five hydrogen bonds.

FIGURE 52D interactions between the receptor and the proposed ligands, 1MP (a), 2MP (b), and 3MP (c).

**Figure:**
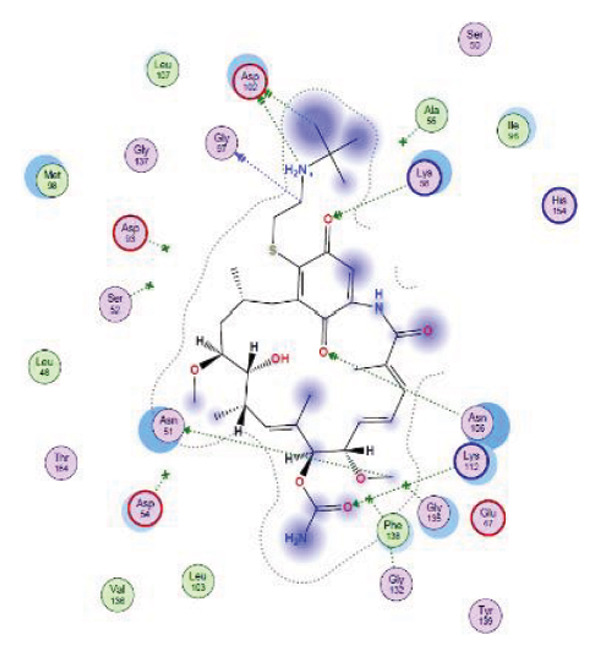
(a)

**Figure:**
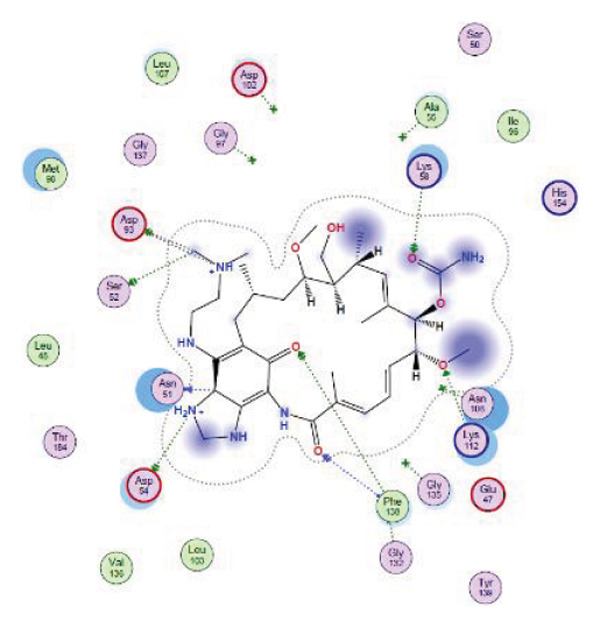
(b)

**Figure:**
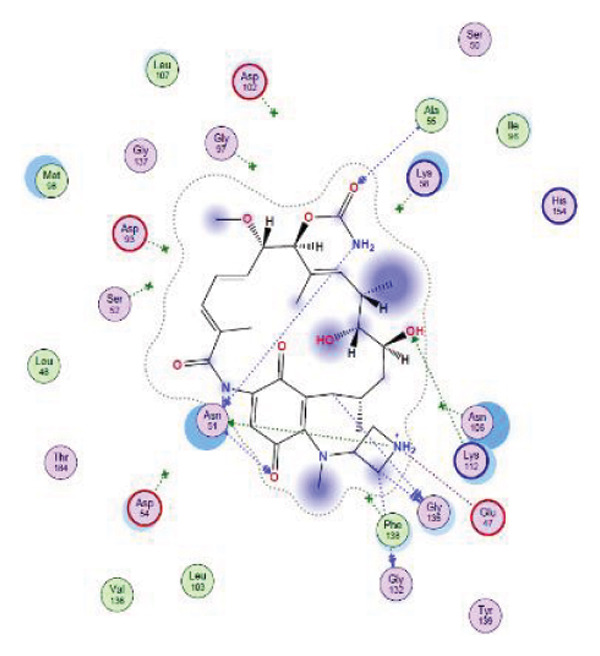
(c)

The first was formed between the carbonyl of the carbamate group at position C7 and the Lys58 residue; the second, with a secondary amine of the imidazolidine ring and the Asp54 residue; the third, between the tertiary amine of the substituent at position C17 and the Asp93 residue; the fourth, between the carbonyl group at position C20 and the Phe130 residue; and the fifth, with the oxygen of the methoxide group at position C6 and the Lys112 residue (Figure 5(b)).

Finally, the 3MP ligand presented a binding energy of −6.74 kcal/mol and formed six hydrogen bonds. The first was established between the secondary amine of the substituent at position C17 and the Glu47 residue; the second, with the oxygen of the hydroxyl group at position C12 and the Lys112 residue; the third, between the carbonyl of the carbamate group at position C7 and the Ala55 residue; and the fourth and fifth involved the primary amine of the carbamate group at the C7 position, the carbonyl group at the C18 position, and the Asp51 residue (Figure 5(c)).

### 3.3. Molecular Dynamics Simulations

The complexes with the best binding energy resulting from the docking were subjected to 100‐ns molecular dynamics simulations to analyze their flexibility and stability over time. The stability of Hsp90 with its ligand co‐crystallized under the same conditions was also simulated to compare the existing conformational and energetic changes using RMSD, RMSF, H‐bond, Rg, and MM‐GBSA calculations.

#### 3.3.1. RMSD

**FIGURE 6:**
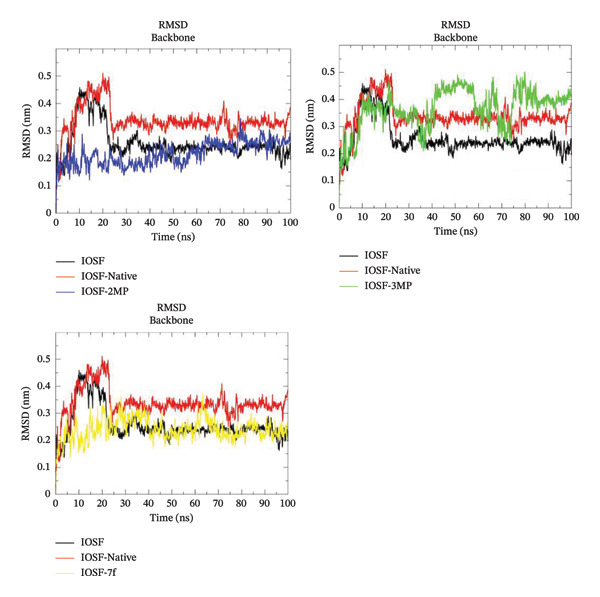
RMSD of protein alone (1OSF) backbone and protein–ligand complexes as a function of time.

The RMSD parameter was calculated, as shown in Figure 6, to evaluate the complexes’ stability and flexibility. The RMSD values range between 0.1 and 0.4 nm, and the protein alone (1OSF) and the native protein–ligand complex show variations between 0.1 and 0.5 nm from the beginning of the simulation until approximately 23 ns, then, in both cases, slight fluctuations occur. On the other hand, ligands 2MP and 7f show minimal fluctuations in this initial interval and maintain small oscillations until the end of the simulation, ligand 3MP presented the most significant number of fluctuations in the molecular dynamic’s simulation; at first, it presents an oscillatory behavior similar to that of the native protein–ligand, reaching a stabilization during the interval of 40 and 60 ns. However, the complex destabilizes after this interval, presenting a much more abrupt movement than with the native ligand or the protein alone.

The RMSD of the ligands (Figure S2) is used to compare the motion of the ligand alone with respect to that of the protein main chain. The native ligand presents a great flexibility in the active site, presenting oscillations between 0.19 nm and 0.1 nm with an average RMSD value of 0.137 ± 0.02 nm. Conversely, Ligand 7f presents fewer oscillations, with only a small change between 40 and 50 ns of the simulation. The average RMSD value is 0.107 ± 0.01 nm, and for the remainder of the simulation, the system remains stable. This suggests that the ligand remains “static” or in an almost constant conformation in the active site. The ligand 2MP showed minimal fluctuations until 15 ns of simulation, where it possibly underwent a conformational change and then stabilized for an interval of 15–55 ns and then returned to its original oscillatory behavior until the end of the simulation, with an average RMSD value of 0.0860 ± 0.02 nm. The ligand 3MP showed the greatest oscillation during the simulation with an average RMSD value of 0.156 ± 0.02 nm.

#### 3.3.2. RMSF

**FIGURE 7:**
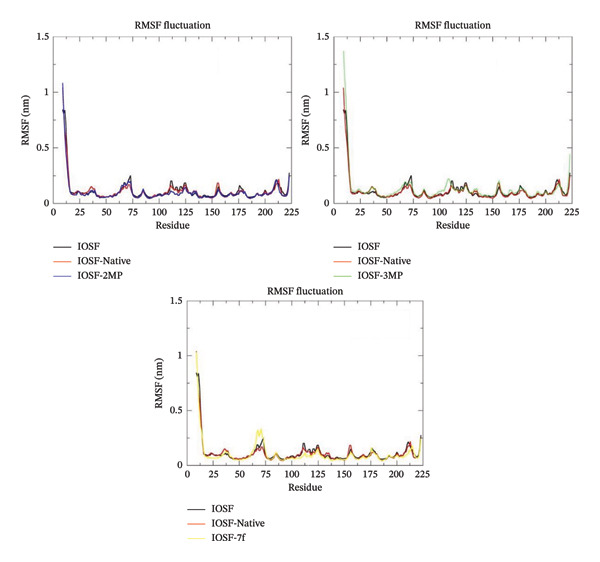
Structural variability (RMSF) of protein alone residues (1OSF) and protein–ligand complexes.

RMSF analysis reveals how ligand binding produces conformational changes in protein residues (Figure 7). Residues with lower RMSF values are more stable, as they exhibit limited motion during the simulation. The N‐terminal end of the protein exhibits the greatest fluctuations due to its exposure to solvent. The second significant peak occurs in helix *α*1, between residues 25 and 34, approximately. Subsequently, a third peak is observed in one of the helices involved in the ligand active site. Helix *α*2, which spans residues 50 to 65, remains relatively stable, which could be attributed to stabilization caused by contact with the ligands. This does not occur in the case of ligand 3MP, as there is a slight increase in fluctuations in this region. Helix *α*3, which spans residues 65 to 76, does not exhibit significant fluctuations.

The area remains stable between residues 76 and 99, corresponding to sheets β2 and β3 and the start of helix *α*4. It is important to note that these last two regions contain the amino acids responsible for the main interactions with ligands. However, from helix *α*4 to residue 130, which includes helices *α*4, *α*5, and *α*6, an increase in fluctuations is observed. The region remains stable from residue 130 to 150, corresponding to helix *α*7, part of the ligand binding site. However, additional peaks are identified between sheets β4 and β7, reaching up to residue 200. Finally, the highest fluctuations are recorded in the residues of the C‐terminal domain, which include helices *α*8 and *α*9 and sheet β8.

#### 3.3.3. Hydrogen Bonds

**FIGURE 8:**
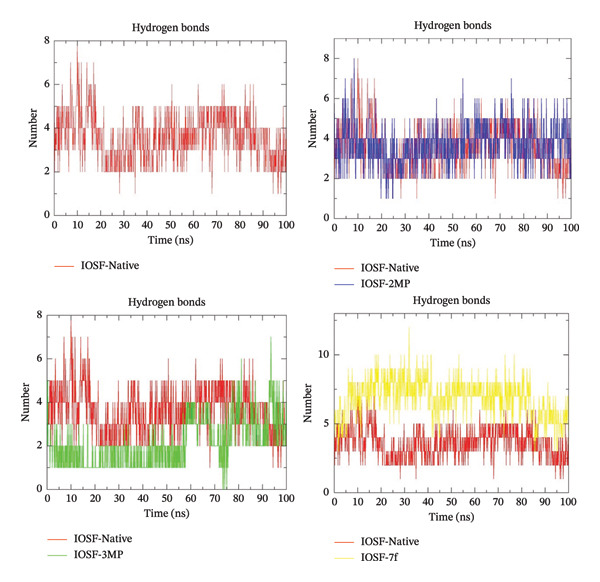
Hydrogen bonds between the protein and the ligands as a function of time.

The number of hydrogen bonds between the protein and the ligand plays a fundamental role in stabilizing protein–ligand complexes (Figure 8) and shows how these interactions vary under dynamic conditions. In docking, the native ligand formed 6 hydrogen bonds. At the same time, molecular dynamics showed an average of 3–4 hydrogen bonds, reaching a maximum of 8, suggesting the formation of transient interactions during the simulation. Ligand 7f, which formed four hydrogen bonds in docking, excelled in dynamics with an average of 6–8 hydrogen bonds and a maximum of 12, demonstrating its ability to maintain additional interactions in the dynamic environment. The ligand 2MP formed 5 hydrogen bonds in docking, but its average dropped to 3–4 in the simulation, reaching a maximum of 8. Finally, the ligand 3MP showed the lowest number of stable interactions, with an average of 2–3 hydrogen bonds and a maximum of 7 in the molecular dynamics.

#### 3.3.4. Solvent‐Accessible Surface Area (SASA)

The SASA describes the variation of the surface area of the protein and the protein–ligand complexes during the simulation period; a high value in this analysis translates into an expansion of the protein. On the other hand, if lower SASA values are obtained, the stability of the complex can increase [36]. The SASA of the protein and the protein–ligand complexes oscillated within a range of Figure S3. The complex that obtained the lowest average of SASA was with the native ligand with 116.48 nm^2^, followed by the proposed 2MP ligand, with 116.61 nm^2^, both of which presented lower SASA values than with the protein alone with an average of 116.82 nm^2^. The third proposed ligand presented slightly larger SASA values with an average of 118.08 nm^2^. The complex with ligand 7f presented the highest average value of 119.20 nm^2^.

#### 3.3.5. Radius of Gyration

The radius of gyration is an important parameter to evaluate the stability of proteins in molecular dynamics simulations. It is defined as the distribution of the atoms of a protein around its axis. The length that represents the distance between the point when it is rotating and the point where the energy transfer has its maximum effect results in the radius of gyration. When a ligand binds to a protein, a conformational change occurs that changes the radius of gyration. In addition, it measures the degree of compaction of the protein and the protein–ligand complexes [37]. The radius of gyration of the protein increases considerably in the absence of a ligand, with an average of 1.78 nm, and this suggests that the degree of compaction and rigidity is much lower. In general, they remain in stable intervals below 0.5 nm, except for ligand 7f, whose radius of gyration exceeds 0.5, with an average of 0.51 nm and a maximum value of 0.53 nm throughout the simulation. The ligand 2MP showed the highest degree of compaction with an average of 0.44 nm (Figure S4).

#### 3.3.6. MM‐GBSA Binding Free Energies Calculation

**FIGURE 9:**
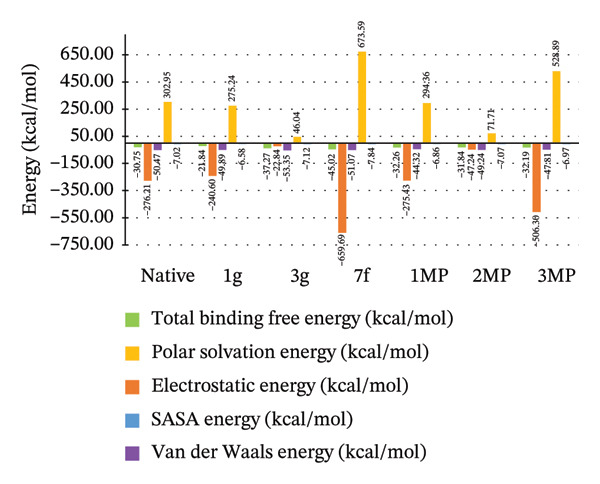
The binding free energy terms obtained from the MM‐GBSA calculations relative to the binding of compounds as labeled in the plot and the color codes for different energy components.

The binding free energy calculation between the protein and the ligands is presented in Figure 9. It is observed that ligands 7f, 3g, 1MP, 2MP, and 3MP showed the most favorable binding free energies, with values of −45.02 kcal/mol, −37.27 kcal/mol, −32.26 kcal/mol, −32.19 kcal/mol, and −31.84 kcal/mol, respectively (see Table S3). According to the results, the electrostatic component was crucial in stabilizing the complexes since this parameter compensated for the polar solvation component’s unfavorable impact, destabilizing the system. On the other hand, although hydrophobic interactions and the SASA component contributed minimally, these interactions also played an essential role in favoring the binding free energy of the complexes.

#### 3.3.7. Pharmacokinetic Properties (ADME)

The pharmacokinetic properties must be evaluated to design a drug delivery form suitable for safe and practical transport [38]. Once the drug enters the body, the body begins to process 4 aspects of the drug: ADME. The pharmacokinetics of a drug predict whether a drug will be able to reach its site of action in its active form. The compounds that were taken as reference are GDM and its derivative 17‐DMAG. One of the main problems that these have presented in their preclinical trials [38, 39] is their low solubility in water, limited oral bioavailability, and the hepatotoxicity they produce [12]. Therefore, the pharmacokinetic properties are predicted to evaluate the effect of the structural modifications and determine if these improve their pharmacological profile and reduce the associated adverse effects. The first assessed parameter in a drug is its balance between lipophilicity and solubility in an aqueous medium, which is shown in Table 8. The lipophilicity of a drug is a measure of how well it dissolves in lipids or a nonpolar solvent. This property is essential since it plays a vital role in the absorption of drugs through membranes or areas of low hydrophilicity [40, 41]. All the proposed ligands showed predicted Log *p* values between 0 and 5, which is considered the optimal range for a drug [42, 43]. On the other hand, solubility plays a fundamental role in the absorption, distribution, and formulation of drugs. To facilitate transport, it is expected that the active ingredients are present in the form of an aqueous solution at the absorption site [44]; 2MP and 3MP presented the highest predicted solubility values, which gives them a good balance in terms of their lipophilicity and solubility. Therefore, it is predicted to have better absorption and distribution than its “precursors” [45, 46]. However, the predicted solubility of 1MP was relatively lower than that of GDM and 17‐DMAG.

**TABLE 8:** Physicochemical properties, lipophilicity, and solubility parameters.

Physicochemical properties					
Compound	17‐DMAG	GMD	1MP	2MP	3MP
Chemical formula	C_32_H_48_N_4_O_8_	C_29_H_40_N_2_O_9_	C_34_H_51_N_3_O_8_S	C_31_H_44_N_4_O_8_	C_34_H_54_N_6_O_7_
MW	616.75	560.64	661.85	600.70	658.83
#Heavy atoms	44	40	46	43	47
#Aromatic heavy atoms	0	0	0	0	0
Fraction Csp^3^	56	52	59	55	62
#Rotatable bonds	8	5	9	5	9
#H‐bond acceptors	9	9	9	9	9
#H‐bond donors	4	3	4	5	6
MR	170.07	151.03	184.41	167.14	190.8

*Lipophilicity parameters*					
TPSA	169.52	163.48	191.58	180.52	176.51
iLOGP	3.16	3.13	3.72	2.96	4.20
XLOGP3	2.04	1.99	3.08	1.01	1.52
WLOGP	1.53	2.03	3.50	2.50	3.00
MLOGP	−7.20	−5.40	4.00	−9.10	−6.20
Silicos‐IT Log P	4.60	7.40	2.29	−4.70	−6.00
Consensus Log P	1.29	1.47	2.60	5.70	1.07

*Water Solubility Parameters*					
ESOL Log S	−4.42	−4.24	−5.29	−3.87	−4.29
ESOL solubility (mg/mL)	2.34	3.23	0.34	8.09	3.39
ESOL solubility (mol/L)	3.79E‐03	5.76E‐03	5.13E‐04	1.35E‐02	5.15E‐03
ESOL class	MS	MS	MS	S	MS
Ali Log S	−5.23	−5.05	−6.77	−4.39	−4.84
Ali solubility (mg/mL)	0.365	0.5	0.0112	2.45	0.963
Ali solubility (mol/L)	5.92E‐04	8.93E‐04	1.70E‐05	4.07E‐03	1.46E‐03
Ali class	MS	MS	PS	MS	MS
Silicos‐IT LogSw	−4.02	−3.25	−5.17	−2.75	−4.84
Silicos‐IT solubility (mg/mL)	5.85	31.3	0.447	108	0.941
Silicos‐IT solubility (mol/L)	9.48E‐03	5.58E‐02	6.76E‐04	1.80E‐01	1.43E‐03
Silicos‐IT class	MS	S	MS	S	MS

Regarding the pharmacokinetic properties (Table 9), it is predicted that there will be no permeability across the blood–brain barrier (BBB), which is not a concern since this drug is not primarily intended for that purpose. On the other hand, P‐glycoprotein is one of the most studied ABC (ATP‐Binding Cassette) transporters. It functions as an efflux transporter for drugs and xenobiotics [47, 48]. This protein plays a fundamental role in drug absorption and excretion and can induce multidrug resistance by limiting the oral absorption of certain drugs, as it pumps them out of the cell, reducing the drug’s effective concentration [49]. It is predicted that all ligands will be substrates of this protein, which poses a challenge, as it has contributed to the low effectiveness of chemotherapy in cancer treatments [42, 50].

**TABLE 9:** Predicted pharmacokinetic parameters.

Pharmacokinetics parameters					
Compound	17‐DMAG	GMD	1MP	2MP	3MP
GI absorption	Low	Low	Low	Low	Low
BBB permeant	No	No	No	No	No
P‐gp substrate	Yes	Yes	Yes	Yes	Yes
CYP1A2 inhibitor	No	No	No	No	No
CYP2C19 inhibitor	No	No	No	No	No
CYP2C9 inhibitor	No	No	No	No	No
CYP2D6 inhibitor	No	No	No	No	No
CYP3A4 inhibitor	No	No	Yes	No	No
Log Kp (cm/s)	−8.6	−8.3	−8.1	−9.2	−9.2

*Drug-likeness, medicinal chemistry, and lead-likeness pharmacokinetics parameters*					
Lipinski #violations	2	2	2	2	3
Ghose #violations	3	3	3	3	3
Veber #violations	1	1	1	1	1
Egan #violations	1	1	1	1	1
Muegge #violations	2	1	2	2	3
Bioavailability Score	17	11	17	17	17
PAINS #alerts	1	1	1	1	0
Brenk #alerts	2	2	2	2	1
Lead‐likeness #violations	2	1	2	1	2
Synthetic Accessibility	8.1	7.7	8.5	7.9	8.6

The interactions between compounds and the cytochrome P450 metabolizing system are essential to understanding the transformations and elimination of the drug from the system. Inhibition of isoforms of this enzyme can result in poor elimination, inducing toxicity to the body [51]. A candidate must have limited inhibitory activity against these compounds. An increase in lipophilicity may enhance the likelihood of drugs becoming substrates for cytochrome enzymes and binding to plasma proteins [52–54]. In addition, they can accumulate in different tissues, such as muscle, when log *p* > 3. None of these compounds are predicted to be inhibitors for cytochromes P450 and their isoforms, except for the ligand 1MP, which is expected to inhibit CYP3A4. Regarding drug‐likeness characteristics, the extent to which the compounds’ physicochemical and structural properties are consistent with most known drugs is evaluated using Lipinski’s Rule of 5 as a reference. It should be noted that most of the compounds studied violate at least two of these rules since they have a molecular weight greater than 500 g/mol, more than five donors, and/or 10 hydrogen bond acceptors. Therefore, it is predicted that they will have a low potential to be absorbed into the systemic circulation by the gastrointestinal tract, like other low molecular weight drugs, as observed in Figure S5. Generally, GDM derivatives are applied parenterally because improving gastrointestinal absorption would imply modifying part of the pharmacophore of GDM and its derivatives with more complex substitutions [6]. The ligand 3MP violates 3 of Lipinski’s rules. Therefore, passive gastrointestinal absorption is predicted to be relatively poor compared to the rest of the compounds [55]. In the ligand 3MP, part of the benzoquinone was altered, which decreased the PAINS and BENKS alerts associated with this compound. Conversely, as observed in the radars, the first proposed ligand and the third one fall within a range of flexibility suitable for a drug, as shown in Figure S6. However, regarding the general characteristics of the ligand 1MP, its pharmacokinetic properties make it less viable for clinical use. Conversely, compounds 2MP and 3MP, such as solubility, slightly improve the characteristics of 17‐DMAG and GDM. However, much more in‐depth and accurate studies must be continued to model a drug that is completely safe for human use.

## 4. Discussion

Assuming that both docking and molecular dynamics assume that the ligand has reached the protein’s active site intact, some ligands with better biological activity than the native ligand and those presented in Table 7 may have higher binding energies. However, the mechanisms these ligands use to reach the cytosol and bind to Hsp90*α* could be more effective than those with higher affinity in the active site. In addition, it has been observed that the reactivity of benzoquinone is sufficient to reduce the viability of a cell culture and that different substituents can increase or decrease this reactivity [56]. On the other hand, the results showed that ligands with a lower affinity energy formed fewer hydrogen bonds than other ligands with a higher affinity energy. One reason may be the conformational change these ligands must acquire to form the obtained interactions; therefore, a ligand can have more interactions depending on the loss of conformational stability.

The QSAR model obtained suggests that the mean inhibitory concentration (IC_50_) increases with the molar refractivity, the fractional hydrophobic surface, the degree of rigidity of the compound, and a more negative value of the HOMO. This implies that, in general, the greater the volume or degree of polarization of a molecule, the lower its activity will be unless these changes favor the total polar surface area (TPSA) of the molecule, which would reduce the fractional hydrophobic surface. In molecular dynamics, it was observed that electrostatic interactions play a crucial role in receptor binding. Substituents of Group 17 usually act as hydrogen bond donors, and an increase in the HOMO energy increases the probability of repulsions between acceptor groups. Furthermore, the model predicts that a higher degree of rigidity in the ligand is associated with lower activity.

Electrostatic energies played a crucial role in ligand–receptor binding during the simulation, as mentioned above. Ligand 7f, which is distinguished from the native ligand by the ring closure at substituent 17 due to the inclusion of a sp^3^ carbon, showed more favorable binding energy. On the other hand, ligand 3g, which features a ring expansion at substituent 17, increased van der Waals–type interactions compared to 7f. On the other hand, the proposed ligands showed a reduction in van der Waals–type energy, as the modeling focused on improving solubility and decreasing the hydrophobic fractional surface area. By comparing the differences between electrostatic and solvation energies, it is observed that ligand 7f induces a greater compensation of the positive contribution of the polar solvation energies in the complex, resulting in a more favorable total binding energy. The proposed ligands show a similar effect, and these could allow the protein to adopt a more electrostatically efficient conformation. Furthermore, the SASA of the 1OSF‐7f complex is, on average, smaller compared to other complexes such as the native one, 3MP, and 1g. This lower solvent exposure, combined with a high proportion of electrostatic interactions, suggests that the ligand could induce a conformation that favors interactions of polar residues with the environment, resulting in the most negative energy values among the set of ligands. It is also observed that some ligands cause a greater degree of motion in the protein. In the case of the 1OSF‐7f complex, although ligand motion is minimal, ligand binding induces a higher protein compaction, as reflected by the radius of gyration (Figure S4). Regarding hydrogen bonds, ligand 7f formed the highest amount, possibly due to the stability of the ligand and the lower mobility of both the ligand and the active site residues. In contrast, ligand 2MP, although forming a lower number of hydrogen bonds on average, contributed to higher complex stability, as observed by the RMSD of both the complex and the ligand.

The QSAR model is based on the activity of compounds evaluated by *in vitro* assays, as mentioned above, and does not consider factors such as absorption, distribution, and metabolism in a specific administration route. For example, one of the parameters of the QSAR model is to reduce the hydrophobicity of the compound. This reduction can be beneficial as it increases the compound’s solubility and reduces the risk of bioaccumulation in tissues with a high affinity for compounds with a high log P. However, pharmacokinetic property predictions suggest that such modifications may decrease gastrointestinal absorption, as shown in Figure S5. In addition, increased ligand flexibility may lead to lower ligand stability. Although gastrointestinal absorption may decrease by adjusting other parameters, these modifications may improve the properties of highly toxic compounds predicted to have higher absorption. For example, 17‐DMAG, a derivative that improves the characteristics of GDM, is expected to have lower gastrointestinal absorption.

## 5. Conclusion

The results indicate that although some ligands exhibit higher binding energies than the 17‐DMAG, their efficiency in reaching the cytosol and interacting with Hsp90*α* may be superior. The QSAR model suggests that inhibitory activity is modulated by molecular descriptors such as molar refractivity, fractional hydrophobic surface area, and compound rigidity. Additionally, electrostatic interactions play a pivotal role in ligand–receptor binding, where structural modifications—such as the incorporation of an sp^3^ carbon—can enhance binding affinity by improving steric complementarity and interaction stability.

The proposed compounds exhibit a reduction in van der Waals energy, aligning with the design strategy aimed at improving aqueous solubility while minimizing hydrophobic interactions. Moreover, these modifications contribute to a more favorable electrostatic profile, promoting interactions with polar residues and potentially stabilizing the receptor–ligand complex. Overall, these findings provide valuable insights for the rational design of next‐generation Hsp90*α* inhibitors with optimized pharmacokinetic and physicochemical properties. However, further experimental validation is necessary to fully characterize their bioavailability and metabolic stability.

## Funding

The authors have nothing to report.

## Conflicts of Interest

The authors declare no conflicts of interest.

## Supporting Information

Additional supporting information can be found online in the Supporting Information section.

## Supporting information


**Supporting Information** Figure S1. Three‐dimensional view of the top five complexes with the receptor (1OSF) and the best ligands: 1g (green), 3g (brown), 7f (yellow), 7a (silver), and 4d (blue). Figure S2. Root mean square deviation of ligands as a function of time. Figure S3. Solvent‐accessible surface area of protein alone and protein–ligand complexes. Figure S4. Radius of gyration of the protein alone and protein–ligand complexes. Figure S5. Egg diagram shows the region in which the ligands used as reference and the ligands studied enter. Figure S6. Pharmacokinetic properties radar. Table S1. RMSD values, energy, and ligand efficiency (EL) obtained in each of the validations. Table S2. Molecular docking results of the proposed ligands. Binding energy and ligand efficiency (EL) are shown. Table S3. Molecular dynamics energy parameters calculated from the MM‐GBSA method.

## Data Availability

The data that support the findings of this study are available from the corresponding author upon reasonable request.
