# Supplementary material for: In Silico Study of 17‐DMAG Derivatives: Integrating QSAR, Molecular Docking, Molecular Dynamics, and ADME Analysis
Source: ScientificWorldJournal. 2026 Jun 17;2026:1136652. doi: 10.1155/tswj/1136652 (PMC13276006; doi:10.1155/tswj/1136652)
Supplement: Supplementary file 1 — Supporting Information Figure S1. Three‐dimensional view of the top five complexes with the receptor (1OSF) and the best ligands: 1g (green), 3g (brown), 7f (yellow), 7a (silver), and 4d (blue). Figure S2. Root mean square deviation of ligands as a function of time. Figure S3. Solvent‐accessible surface area of protein alone and protein–ligand complexes. Figure S4. Radius of gyration of the protein alone and protein–ligand complexes. Figure S5. Egg diagram shows the region in which the ligands used as reference and the ligands studied enter. Figure S6. Pharmacokinetic properties radar. Table S1. RMSD values, energy, and ligand efficiency (EL) obtained in each of the validations. Table S2. Molecular docking results of the proposed ligands. Binding energy and ligand efficiency (EL) are shown. Table S3. Molecular dynamics energy parameters calculated from the MM‐GBSA method. [file TSWJ-2026-1136652-s001.docx]

**INFORMATION SUPPORT**

**7a**

**-7.28 kcal/mol**

**4d**

**-7.25 kcal/mol**

**1g**

**3g**

**7f**


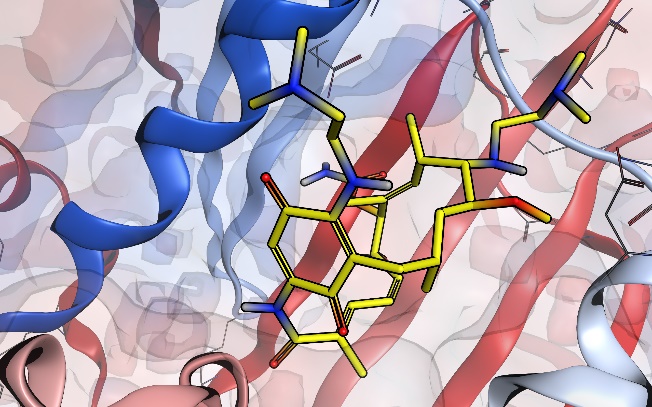

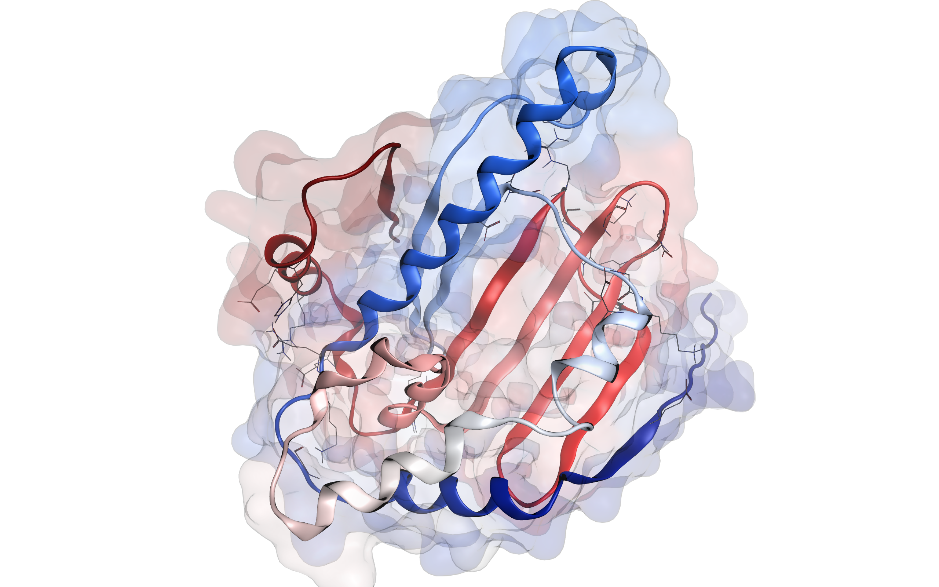


C-terminus

N-terminus


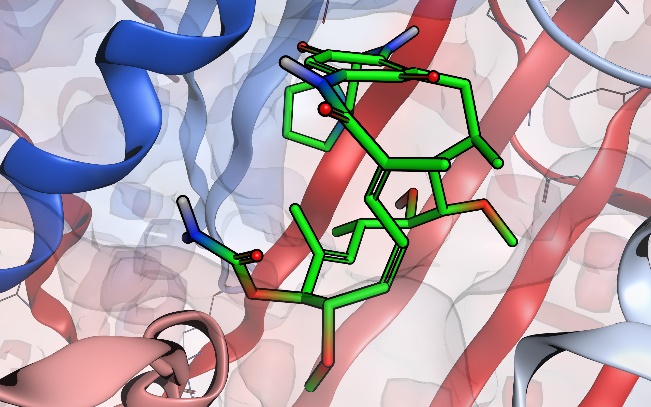

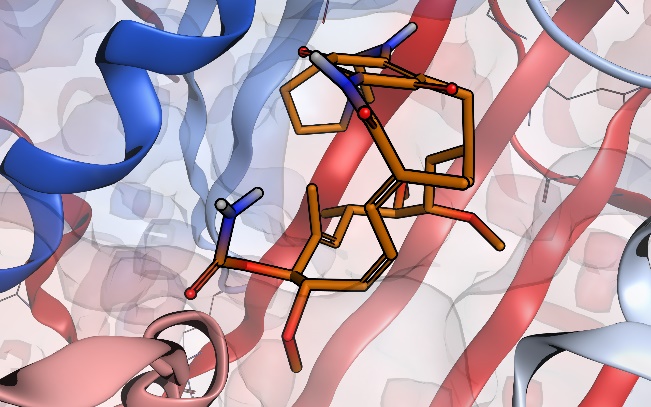


**-7.81 kcal/mol**

**-7.50 kcal/mol**

**-7.32 kcal/mol**

Figure S1. Three-dimensional view of the top five complexes with the receptor (1OSF) and the best ligands: 1g (green), 3g (brown), 7f (yellow), 7a (silver), and 4d (blue)


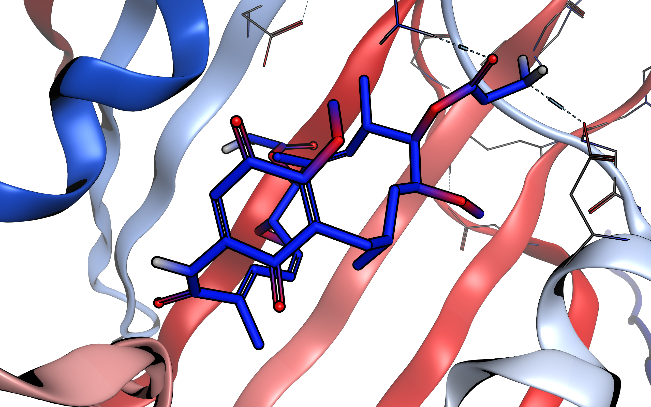

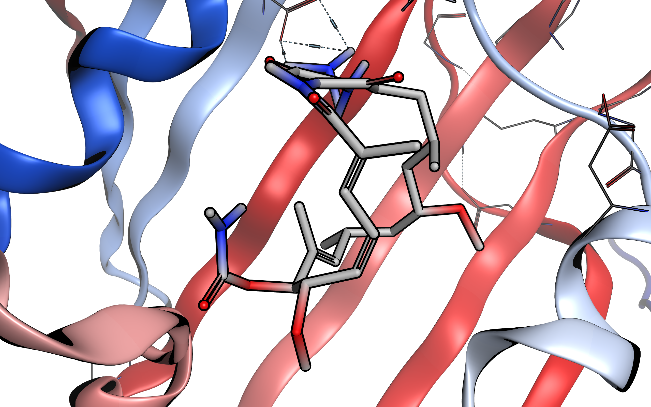

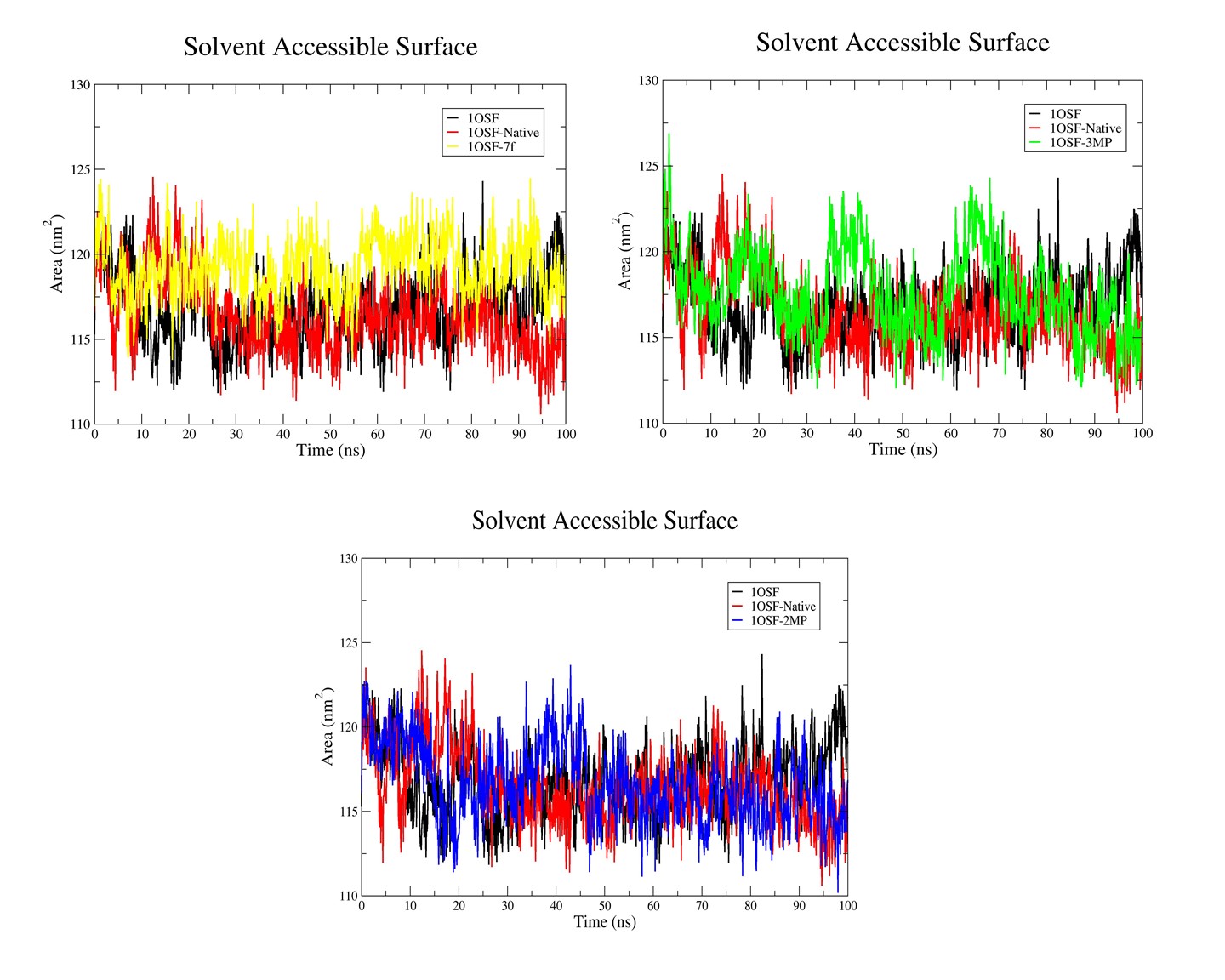


Figure S3. Solvent-accessible surface area of ​​protein alone and protein-ligand complexes.

Figure S2. Root mean square deviation of ligands as a function of time.


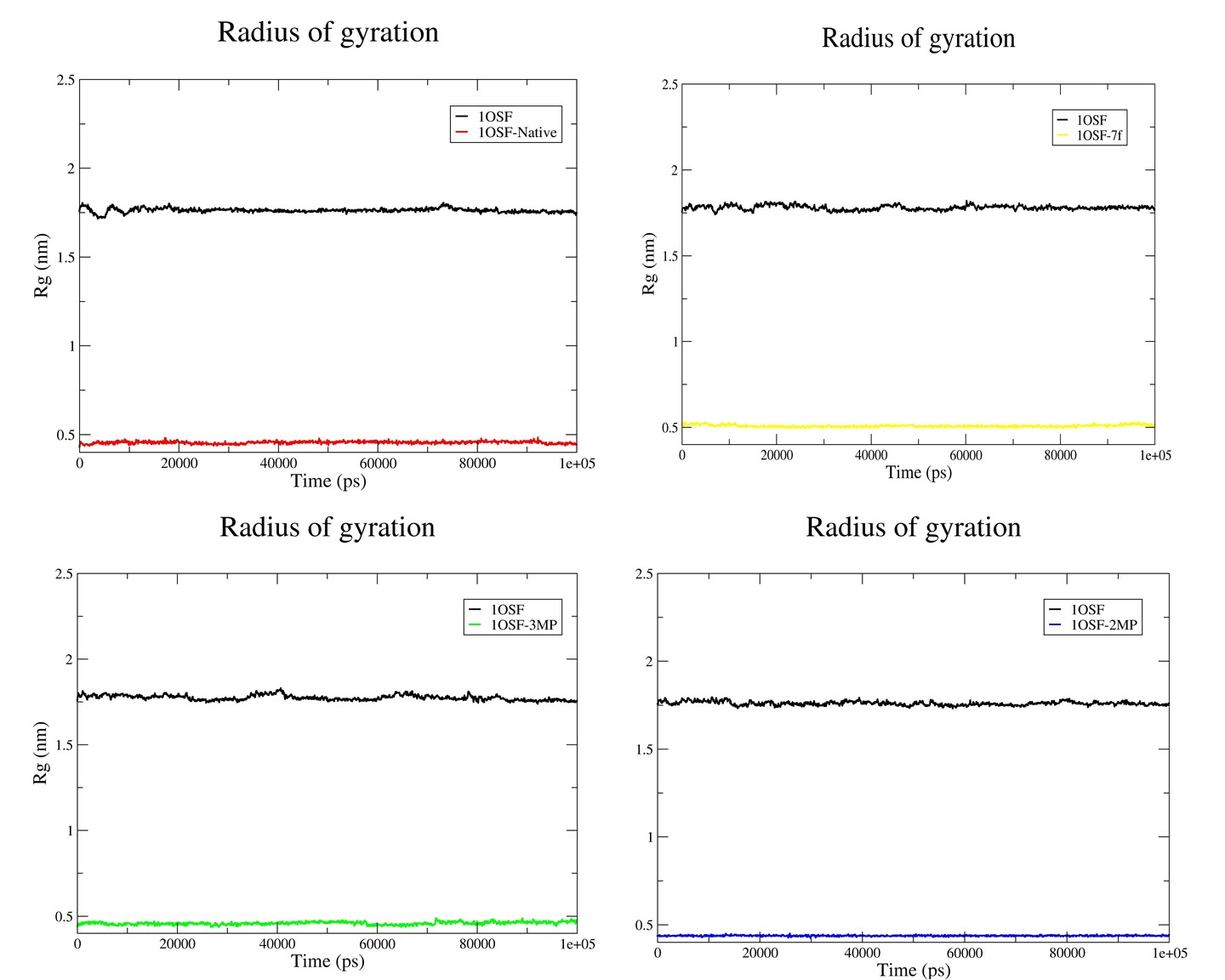


Figure S4. Radius of gyration of the protein alone and protein-ligand complexes


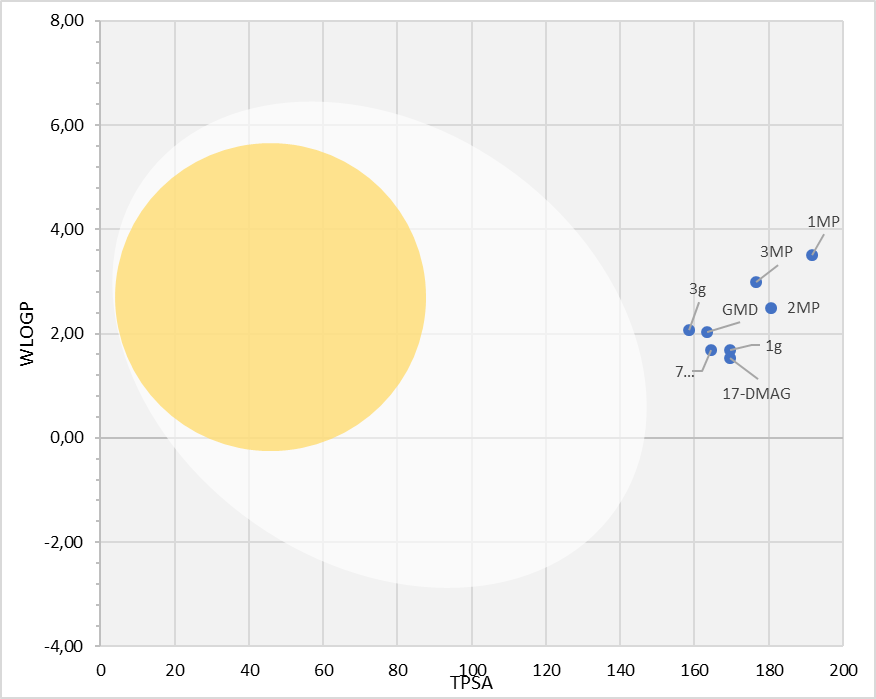


Figure S5. Egg diagram. Shows the region in which the ligands used as reference and the ligands studied enter.

| **NATIVE** | **1g** | **3g** |
| --- | --- | --- |
| 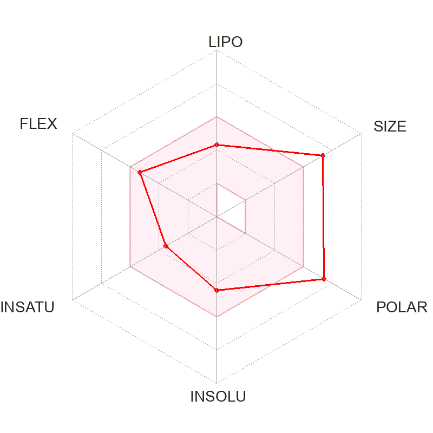 | 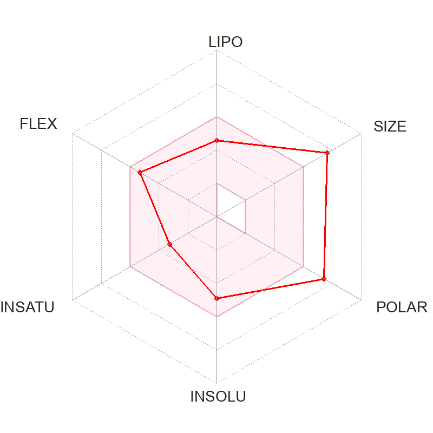 | 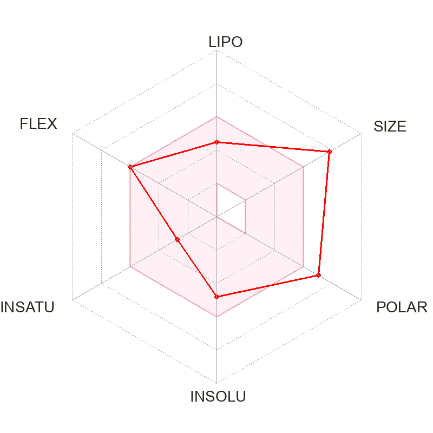 |
| **7f** | **1MP** | **2MP** |
| 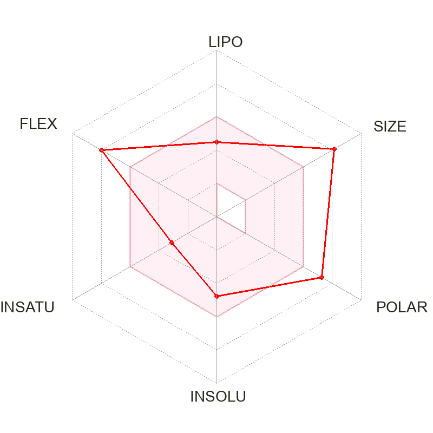 | 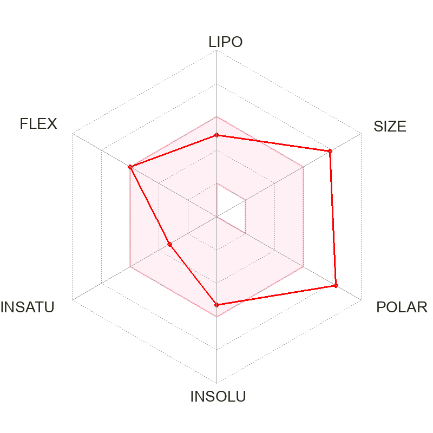 | 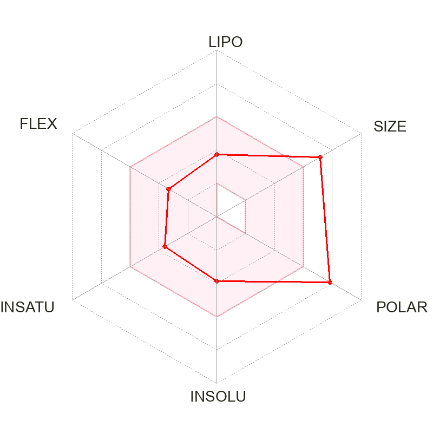 |
|  | **3MP** |  |
|  | 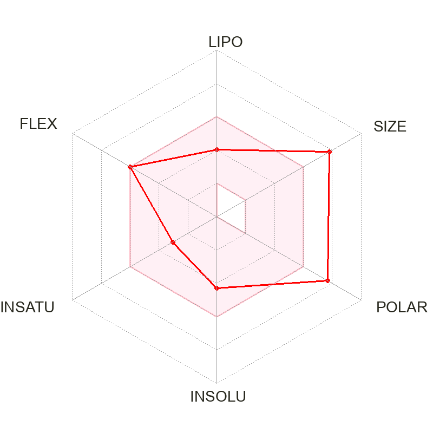 |  |
| Figure S6. Pharmacokinetic properties radar. | | |

Table S1. RMSD values, energy and ligand efficiency (EL) obtained in each of the validations.

| Validation | | | |
| --- | --- | --- | --- |
| Run | RMSD(Å) | Energy (kcal/mol) | EL |
| 1 | 1.002 | -10.11 | -0.23 |
| 2 | 1.140 | -9.83 | -0.23 |
| 3 | 1.142 | -8.43 | -0.19 |
| Average Value | 1.095 ± 0.066 | -9.46 ± 0.735 | -0.217 ± 0.019 |

Table S2. Molecular docking results of the proposed ligands. Binding energy and ligand efficiency (EL) are shown.

| Compounds | Energy (kcal/mol) | EL |
| --- | --- | --- |
| 1MP | -6.35 | -0.15 |
| 2MP | -7.14 | -0.17 |
| 3MP | -6.74 | -0.14 |

Tabla S3. Molecular dynamics energy parameters calculated from the MMGBSA method.

| Energy components | Native | 1g | 3g | 7f | 1MP | 2MP | 3MP |
| --- | --- | --- | --- | --- | --- | --- | --- |
| Total Binding Free Energy (kcal/mol) | -30.75 | -21.84 | -37.27 | -45.02 | -32.26 | -31.84 | -32.19 |
| Electrostatic Energy (kcal/mol) | -276.21 | -240.60 | -22.84 | -659.69 | -275.43 | -47.24 | -506.30 |
| Van der Waals Energy(kcal/mol) | -50.47 | -49.89 | -53.35 | -51.07 | -44.32 | -49.24 | -47.81 |
| Polar solvation Energy (kcal/mol) | 302.95 | 275.24 | 46.04 | 673.59 | 294.36 | 71.71 | 528.89 |
| SASA Energy (kcal/mol) | -7.02 | -6.58 | -7.12 | -7.84 | -6.86 | -7.07 | -6.97 |
